# Supplementary material for: The incidence of admission ionised hypocalcaemia in paediatric major trauma—A systematic review and meta-analysis
Source: PLoS One. 2024 May 28;19(5):e0303109. doi: 10.1371/journal.pone.0303109 (PMC11132510; doi:10.1371/journal.pone.0303109)
Supplement: S1 File — (PDF) [file pone.0303109.s002.pdf]

Online Supplement 2.

The Incidence of Admission Ionised Hypocalcaemia in Paediatric Major Trauma – a Systematic Review and Meta-Analysis

Search Strategy and Results

Number of articles retrieved

Database	Total retrieved	Total included
Medline	6	6
CINAHL	4	2
EMBASE	12	7

Total included from all databases	22
Total duplicates	7
Total included in the results	15

Limiters

English language

Other limiters

Human, patients under 16

CINAHL

Search ID#	Search Terms	Last Run Via	Results
S29	S25 AND S28	Interface - EBSCOhost Research Databases<br>Search Screen - Advanced Search<br>Database - CINAHL	2
S28	S26 OR S27	Interface - EBSCOhost Research Databases<br>Search Screen - Advanced Search<br>Database - CINAHL	95,090
S27	AB trauma	Interface - EBSCOhost Research Databases<br>Search Screen - Advanced Search<br>Database - CINAHL	77,482
S26	TI trauma	Interface - EBSCOhost Research Databases<br>Search Screen - Advanced Search<br>Database - CINAHL	39,417
S25	S23 OR S24	Interface - EBSCOhost Research Databases<br>Search Screen - Advanced	11

Online Supplement 2.

The Incidence of Admission Ionised Hypocalcaemia in Paediatric Major Trauma – a Systematic Review and Meta-Analysis

		Search<br>Database - CINAHL	
S24	AB paediatric hypocalcaemia or pediatric hypocalcemia	Interface - EBSCOhost Research Databases<br>Search Screen - Advanced Search<br>Database - CINAHL	2
S23	TI paediatric hypocalcaemia or pediatric hypocalcemia	Interface - EBSCOhost Research Databases<br>Search Screen - Advanced Search<br>Database - CINAHL	9
S22	S3 AND S17	Interface - EBSCOhost Research Databases<br>Search Screen - Advanced Search<br>Database - CINAHL	351
S21	S3 AND S12	Interface - EBSCOhost Research Databases<br>Search Screen - Advanced Search<br>Database - CINAHL	0
S20	S18 OR S19	Interface - EBSCOhost Research Databases<br>Search Screen - Advanced Search<br>Database - CINAHL	19
S19	AB admission hypocalcaemia or admission hypocalcemia	Interface - EBSCOhost Research Databases<br>Search Screen - Advanced Search<br>Database - CINAHL	18
S18	TI admission hypocalcaemia or admission hypocalcemia	Interface - EBSCOhost Research Databases<br>Search Screen - Advanced Search<br>Database - CINAHL	1
S17	S15 OR S16	Interface - EBSCOhost Research Databases<br>Search Screen - Advanced Search<br>Database - CINAHL	835,597
S16	AB outcomes	Interface - EBSCOhost Research Databases<br>Search Screen - Advanced Search<br>Database - CINAHL	732,991

Online Supplement 2.

The Incidence of Admission Ionised Hypocalcaemia in Paediatric Major Trauma – a Systematic Review and Meta-Analysis

S15	TI outcomes	Interface - EBSCOhost Research Databases<br>Search Screen - Advanced Search<br>Database - CINAHL	213,098
S14	S3 AND S9 AND S12	Interface - EBSCOhost Research Databases<br>Search Screen - Advanced Search<br>Database - CINAHL	0
S13	S3 AND S6	Interface - EBSCOhost Research Databases<br>Search Screen - Advanced Search<br>Database - CINAHL	2
S12	S10 OR S11	Interface - EBSCOhost Research Databases<br>Search Screen - Advanced Search<br>Database - CINAHL	83
S11	AB normocalcaemia or normocalcemia	Interface - EBSCOhost Research Databases<br>Search Screen - Advanced Search<br>Database - CINAHL	78
S10	TI normocalcaemia or normocalcemia	Interface - EBSCOhost Research Databases<br>Search Screen - Advanced Search<br>Database - CINAHL	8
S9	S7 OR S8	Interface - EBSCOhost Research Databases<br>Search Screen - Advanced Search<br>Database - CINAHL	1,406
S8	AB hospital arrival	Interface - EBSCOhost Research Databases<br>Search Screen - Advanced Search<br>Database - CINAHL	1,349
S7	TI hospital arrival	Interface - EBSCOhost Research Databases<br>Search Screen - Advanced Search<br>Database - CINAHL	107
S6	S4 OR S5	Interface - EBSCOhost Research Databases	1,745

Online Supplement 2.

The Incidence of Admission Ionised Hypocalcaemia in Paediatric Major Trauma – a Systematic Review and Meta-Analysis

		Search Screen - Advanced Search<br>Database - CINAHL	
S5	AB hypocalcaemia or hypocalcemia	Interface - EBSCOhost Research Databases<br>Search Screen - Advanced Search<br>Database - CINAHL	1,451
S4	TI hypocalcaemia or hypocalcemia	Interface - EBSCOhost Research Databases<br>Search Screen - Advanced Search<br>Database - CINAHL	584
S3	S1 OR S2	Interface - EBSCOhost Research Databases<br>Search Screen - Advanced Search<br>Database - CINAHL	1,003
S2	AB paediatric trauma patients or pediatric trauma patients	Interface - EBSCOhost Research Databases<br>Search Screen - Advanced Search<br>Database - CINAHL	805
S1	TI paediatric trauma patients or pediatric trauma patients	Interface - EBSCOhost Research Databases<br>Search Screen - Advanced Search<br>Database - CINAHL	364

Online Supplement 2.

The Incidence of Admission Ionised Hypocalcaemia in Paediatric Major Trauma – a Systematic Review and Meta-Analysis

MEDLINE

Search ID#	Search Terms	Last Run Via	Results
S30	S26 AND S29	Interface - EBSCOhost Research Databases<br>Search Screen - Advanced Search<br>Database - MEDLINE	5
S29	S27 OR S28	Interface - EBSCOhost Research Databases<br>Search Screen - Advanced Search<br>Database - MEDLINE	276,599
S28	AB trauma	Interface - EBSCOhost Research Databases<br>Search Screen - Advanced Search<br>Database - MEDLINE	245,191
S27	TI trauma	Interface - EBSCOhost Research Databases<br>Search Screen - Advanced Search<br>Database - MEDLINE	87,100
S26	S24 OR S25	Interface - EBSCOhost Research Databases<br>Search Screen - Advanced Search<br>Database - MEDLINE	38
S25	AB paediatric hypocalcaemia or pediatric hypocalcemia	Interface - EBSCOhost Research Databases<br>Search Screen - Advanced Search<br>Database - MEDLINE	22
S24	TI paediatric hypocalcaemia or pediatric hypocalcemia	Interface - EBSCOhost Research Databases<br>Search Screen - Advanced Search<br>Database - MEDLINE	18
S23	S6 AND S22	Interface - EBSCOhost Research Databases<br>Search Screen - Advanced Search<br>Database - MEDLINE	4
S22	S3 AND S17	Interface - EBSCOhost Research Databases<br>Search Screen - Advanced	746

Online Supplement 2.

The Incidence of Admission Ionised Hypocalcaemia in Paediatric Major Trauma – a Systematic Review and Meta-Analysis

		Search<br>Database - MEDLINE	
S21	S3 AND S12	Interface - EBSCOhost Research Databases<br>Search Screen - Advanced Search<br>Database - MEDLINE	0
S20	S18 OR S19	Interface - EBSCOhost Research Databases<br>Search Screen - Advanced Search<br>Database - MEDLINE	72
S19	AB admission hypocalcaemia or admission hypocalcemia	Interface - EBSCOhost Research Databases<br>Search Screen - Advanced Search<br>Database - MEDLINE	71
S18	TI admission hypocalcaemia or admission hypocalcemia	Interface - EBSCOhost Research Databases<br>Search Screen - Advanced Search<br>Database - MEDLINE	4
S17	S15 OR S16	Interface - EBSCOhost Research Databases<br>Search Screen - Advanced Search<br>Database - MEDLINE	2,179,197
S16	AB outcomes	Interface - EBSCOhost Research Databases<br>Search Screen - Advanced Search<br>Database - MEDLINE	2,053,450
S15	TI outcomes	Interface - EBSCOhost Research Databases<br>Search Screen - Advanced Search<br>Database - MEDLINE	450,999
S14	S3 AND S9 AND S12	Interface - EBSCOhost Research Databases<br>Search Screen - Advanced Search<br>Database - MEDLINE	0
S13	S3 AND S6	Interface - EBSCOhost Research Databases<br>Search Screen - Advanced Search<br>Database - MEDLINE	5

Online Supplement 2.

The Incidence of Admission Ionised Hypocalcaemia in Paediatric Major Trauma – a Systematic Review and Meta-Analysis

S12	S10 OR S11	Interface - EBSCOhost Research Databases<br>Search Screen - Advanced Search<br>Database - MEDLINE	870
S11	AB normocalcaemia or normocalcemia	Interface - EBSCOhost Research Databases<br>Search Screen - Advanced Search<br>Database - MEDLINE	837
S10	TI normocalcaemia or normocalcemia	Interface - EBSCOhost Research Databases<br>Search Screen - Advanced Search<br>Database - MEDLINE	52
S9	S7 OR S8	Interface - EBSCOhost Research Databases<br>Search Screen - Advanced Search<br>Database - MEDLINE	80,231
S8	AB hospital arrival or hospital admission	Interface - EBSCOhost Research Databases<br>Search Screen - Advanced Search<br>Database - MEDLINE	76,431
S7	TI hospital arrival or hospital admission	Interface - EBSCOhost Research Databases<br>Search Screen - Advanced Search<br>Database - MEDLINE	8,797
S6	S4 OR S5	Interface - EBSCOhost Research Databases<br>Search Screen - Advanced Search<br>Database - MEDLINE	11,987
S5	AB hypocalcaemia or hypocalcemia	Interface - EBSCOhost Research Databases<br>Search Screen - Advanced Search<br>Database - MEDLINE	10,707
S4	TI hypocalcaemia or hypocalcemia	Interface - EBSCOhost Research Databases<br>Search Screen - Advanced Search<br>Database - MEDLINE	3,153
S3	S1 OR S2	Interface - EBSCOhost Research Databases	2,051

Online Supplement 2.

The Incidence of Admission Ionised Hypocalcaemia in Paediatric Major Trauma – a Systematic Review and Meta-Analysis

		Search Screen - Advanced Search<br>Database - MEDLINE	
S2	AB paediatric trauma patients or pediatric trauma patients	Interface - EBSCOhost Research Databases<br>Search Screen - Advanced Search<br>Database - MEDLINE	1,764
S1	TI paediatric trauma patients or pediatric trauma patients	Interface - EBSCOhost Research Databases<br>Search Screen - Advanced Search<br>Database - MEDLINE	649

Online Supplement 2.

The Incidence of Admission Ionised Hypocalcaemia in Paediatric Major Trauma – a Systematic Review and Meta-Analysis

EMBASE

#	Query	Results from 3 Jul 2023
1	(paediatric trauma patients or pediatric trauma patients).m_titl.	384
2	(paediatric trauma patients or pediatric trauma patients).mp. [mp=title, abstract, heading word, drug trade name, original title, device manufacturer, drug manufacturer, device trade name, keyword heading word, floating subheading word, candidate term word]	1,172
3	limit 2 to abstracts	1,155
4	1 or 3	1,172
5	(hypocalcaemia or hypocalcemia).m_titl.	3,811
6	(hypocalcaemia or hypocalcemia).mp. [mp=title, abstract, heading word, drug trade name, original title, device manufacturer, drug manufacturer, device trade name, keyword heading word, floating subheading word, candidate term word]	31,406
7	limit 6 to abstracts	26,928
8	5 or 7	27,890
9	(hospital arrival or hospital admission).m_titl.	4,381
10	(hospital arrival or hospital admission).mp. [mp=title, abstract, heading word, drug trade name, original title, device manufacturer, drug manufacturer, device trade name, keyword heading word, floating subheading word, candidate term word]	292,976
11	limit 10 to abstracts	245,800
12	9 or 11	246,388
13	(normocalcaemia or normocalcemia).m_titl.	63
14	(normocalcaemia or normocalcemia).mp. [mp=title, abstract, heading word, drug trade name, original title, device manufacturer, drug manufacturer, device trade name, keyword heading word, floating subheading word, candidate term word]	1,223
15	limit 14 to abstracts	1,213
16	13 or 15	1,223
17	4 and 8 and 12	1
18	4 and 8	6
19	(paediatric hypocalcaemia or pediatric hypocalcemia).m_titl.	2
20	(paediatric hypocalcaemia or pediatric hypocalcemia).mp. [mp=title, abstract, heading word, drug trade name, original title, device manufacturer, drug manufacturer, device trade name, keyword heading word, floating subheading word, candidate term word]	4
21	limit 20 to abstracts	2
22	19 or 21	4
23	(admission hypocalcaemia or admission hypocalcemia).m_titl.	2
24	(admission hypocalcaemia or admission hypocalcemia).mp. [mp=title, abstract, heading word, drug trade name, original title, device manufacturer, drug manufacturer, device trade	11

Online Supplement 2.

The Incidence of Admission Ionised Hypocalcaemia in Paediatric Major Trauma – a Systematic Review and Meta-Analysis

	name, keyword heading word, floating subheading word, candidate term word]	
25	limit 24 to abstracts	11
26	23 or 25	11
27	4 and 16	0
