# Supplementary material for: The incidence of admission ionised hypocalcaemia in paediatric major trauma—A systematic review and meta-analysis
Source: PLoS One. 2024 May 28;19(5):e0303109. doi: 10.1371/journal.pone.0303109 (PMC11132510; doi:10.1371/journal.pone.0303109)

Online Supplement 4.

The Incidence of Admission Ionised Hypocalcaemia in Paediatric Major Trauma – a Systematic Review and Meta-Analysis

Additional Statistical Analysis

S1: Additional meta-analysis results: Mortality

S2: Additional meta-analysis results: pH difference

Online Supplement 4.

The Incidence of Admission Ionised Hypocalcaemia in Paediatric Major Trauma – a Systematic Review and Meta-Analysis

S1: Additional meta-analysis results: Mortality

Sensitivity to model selection

The detailed results below depict meta-analysis results for the endpoint of mortality. The table information assesses for results sensitivity to random-effects model selection (with DerSimonian-Laird results presented for comparison to the main Sidik-Jonkman results). The table S1-T1 also presents results in fixed-effect modeling, which are presented as an indicator of the degree of heterogeneity. Results of the DerSimonian-Laird random-effects model, and of the fixed-effect model, were not inconsistent with the results from the primary *a priori* modeling method (Sidik-Jonkman). The findings support conclusions of lack of results sensitivity to model selection, and also do not indicate concerning heterogeneity.

Table S1-T1. Morality endpoint: results for assessment of sensitivity to model selection

**Model**	I^2	**Mortality* (95% CI)**	***p*, Cochrane's Q**	***p*, pooled effect departure from null**
DerSimonian-Laird RE (OR metric)	18%	2.01 (0.89-4.57)	.294	.094
Fixed-effect (OR metric)	18%	1.80 (0.95-3.42)	.072	.294

*Numbers exceeding 1.0 correspond to higher mortality with hypocalcaemia

Abbreviations: CI – confidence interval; RE – random effects; OR – odds ratio

Galbraith plot

Galbraith plot (Figure S1-F1) for the endpoint indicated neither heterogeneity nor study outliers.

Online Supplement 4.

The Incidence of Admission Ionised Hypocalcaemia in Paediatric Major Trauma – a Systematic Review and Meta-Analysis

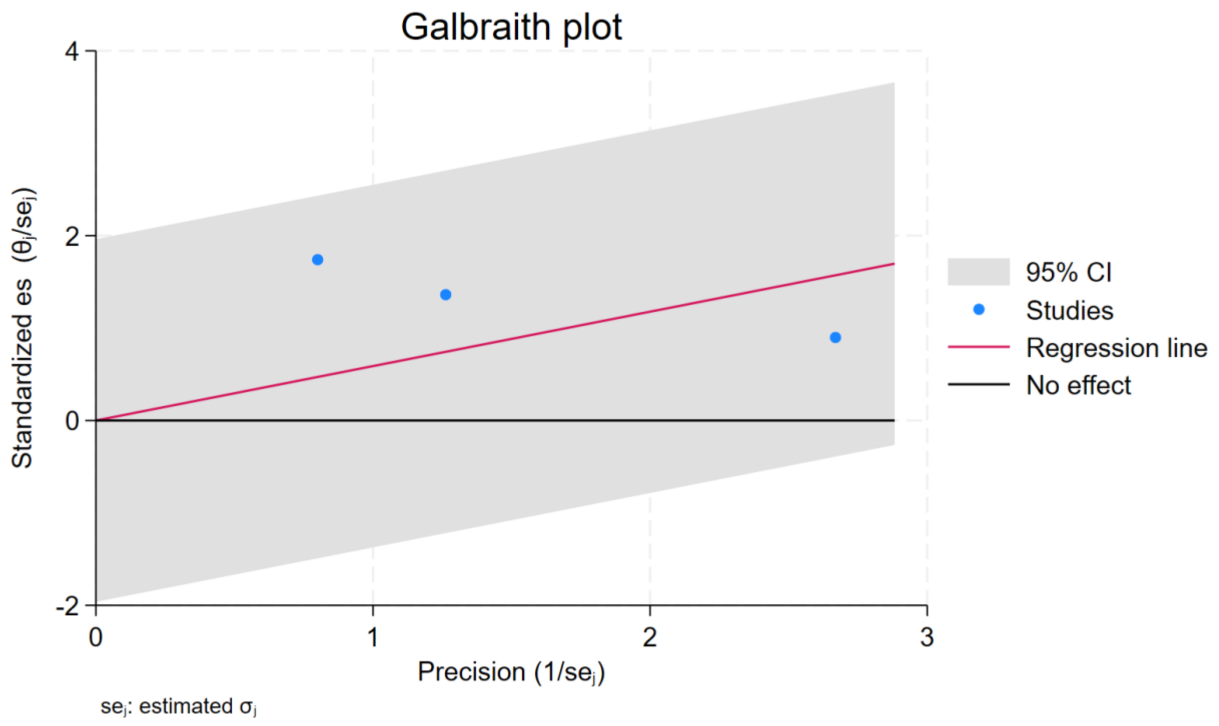

Figure S1-F1. Galbraith plot for the endpoint of mortality

Trim-fill analysis and funnel plotting

The funnel plot for the endpoint is shown in Figure S1-F2. The contoured funnel plot suggested possibility of some degree of small-study bias, but not likely to a degree that substantially affected results. Two studies were imputed, neither with projected statistically significant findings.

With only three studies, there was insufficient study *N* to enable formal funnel asymmetry analysis. However, the mortality OR estimate (1.40) of the observed+imputed studies had a 95% CI (0.44-4.51) that continued to overlap the null finding (of 1.0).

Figure S1-F2. Contoured funnel plot for the endpoint of mortality

Online Supplement 4.

The Incidence of Admission Ionised Hypocalcaemia in Paediatric Major Trauma – a Systematic Review and Meta-Analysis

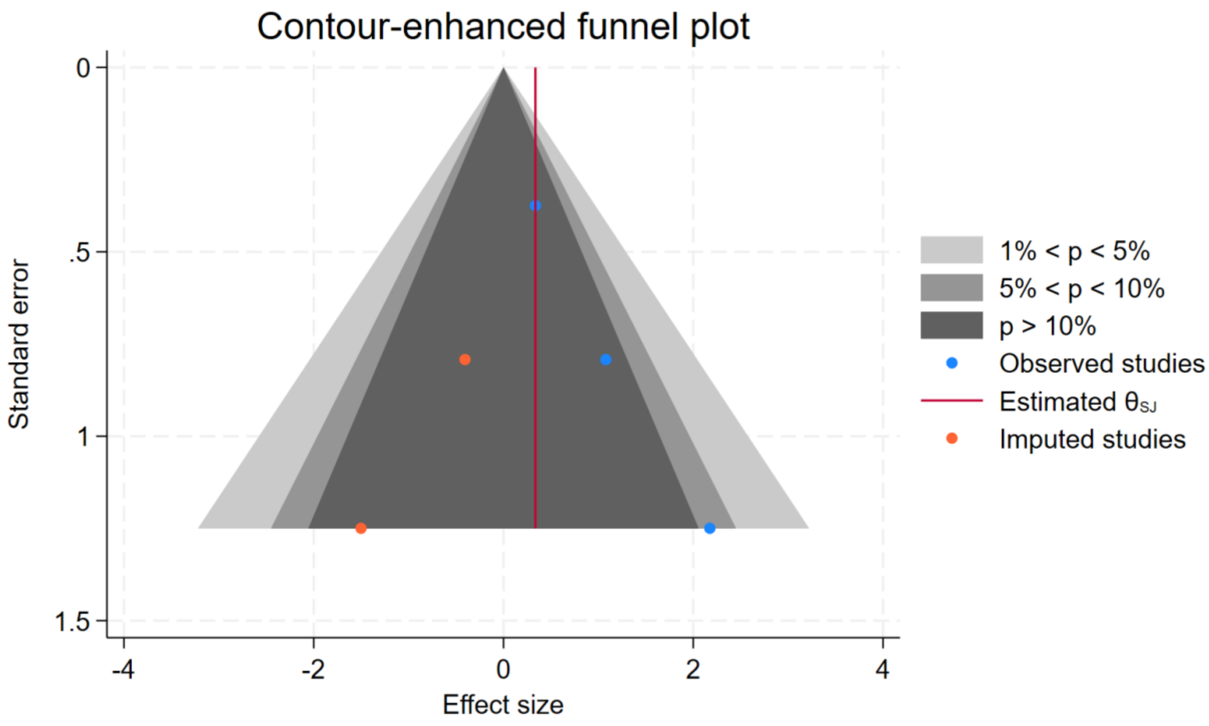

Cumulative meta-analysis

Cumulative meta-analysis for the endpoint was executed ordering studies based on increasing *n* (Figure S1-F3). The smallest *n* (Gimelraikh) commenced the cumulative meta-analysis with a non-significant indicator of higher mortality in hypocalceamics; larger studies indicated a lower point estimate for hypocalcaemia-associated increased mortality but findings continued to be non-significant.

Figure S1-F3. Cumulative analysis for the endpoint of mortality

Online Supplement 4.

The Incidence of Admission Ionised Hypocalcaemia in Paediatric Major Trauma – a Systematic Review and Meta-Analysis

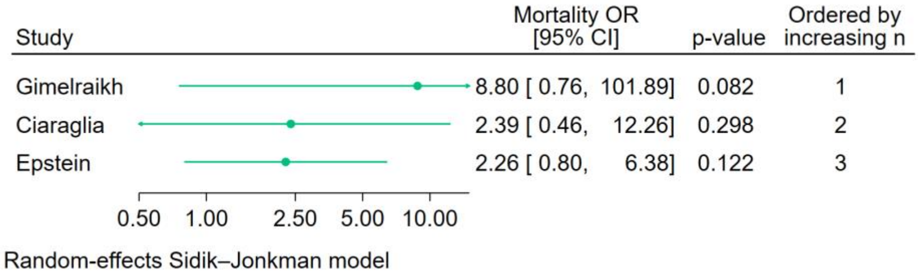

Omitted-study meta-analysis

To ascertain the degree to which individual study results executed inordinate leverage on the overall results, omitted-study (leave-one-out) meta-analysis was executed. The plot in Figure S1-F4 shows the effect estimate that would be generated if the named study were not included in the calculations. The actual overall effect estimate from all studies (*i.e.* as reported in main results) is depicted with a dashed line. The omitted-study analysis for the endpoint suggested that if the Ciaraglia study were dropped from meta-analysis the findings for the effect estimate would approach statistical significance (*p* would equal 0.05). Omission of Ciaraglia would also double the OR from the actually calculated (*i.e.* from all three studies) pooled estimate.

Figure S1-F4. Omitted-study analysis for the endpoint of mortality

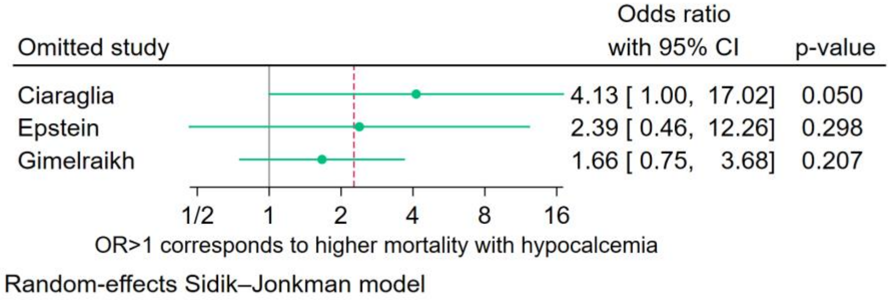

Online Supplement 4.

The Incidence of Admission Ionised Hypocalcaemia in Paediatric Major Trauma – a Systematic Review and Meta-Analysis

S2: Additional meta-analysis results: pH difference

Sensitivity to model selection

The detailed results below depict meta-analysis results for the endpoint of pH difference. The table S2-T1 information assesses for results sensitivity to random-effects model selection (with DerSimonian-Laird results presented for comparison to the main Sidik-Jonkman results). The table also presents results in fixed-effect modeling, which are presented as an indicator of the degree of heterogeneity. Results of the DerSimonian-Laird random-effects model, and of the fixed-effect model, were not inconsistent with the results from the primary *a priori* modeling method (Sidik-Jonkman). The findings support conclusions of lack of results sensitivity to model selection, and also do not indicate concerning heterogeneity.

Table S2-T1: pH difference endpoint: results for assessment of sensitivity to model selection

Model	I^2	pH difference* (95% CI)	*p*, Cochrane's Q	*p*, pooled effect departure from null
DerSimonian-Laird RE (Glass D mean difference)	57%	-0.080 (-0.432 to 0.272)	.010	.656
Fixed-effect (Glass D mean difference)	57%	-0.120 (-0.347 to 0.107)	.010	.301
Sidik-Jonkman RE non-standardized mean difference	70%	-.010 (-0.056 to 0.035)	.068	.657

*Negative numbers correspond to lower pH with hypocalcaemia

Abbreviations: CI – confidence interval; RE – random effects

Galbraith plot

Galbraith plot (Figure S2-F1) for the endpoint indicated neither heterogeneity nor study outliers.

Online Supplement 4.

The Incidence of Admission Ionised Hypocalcaemia in Paediatric Major Trauma – a Systematic Review and Meta-Analysis

Figure S2-F1. Galbraith plot for the endpoint of pH difference

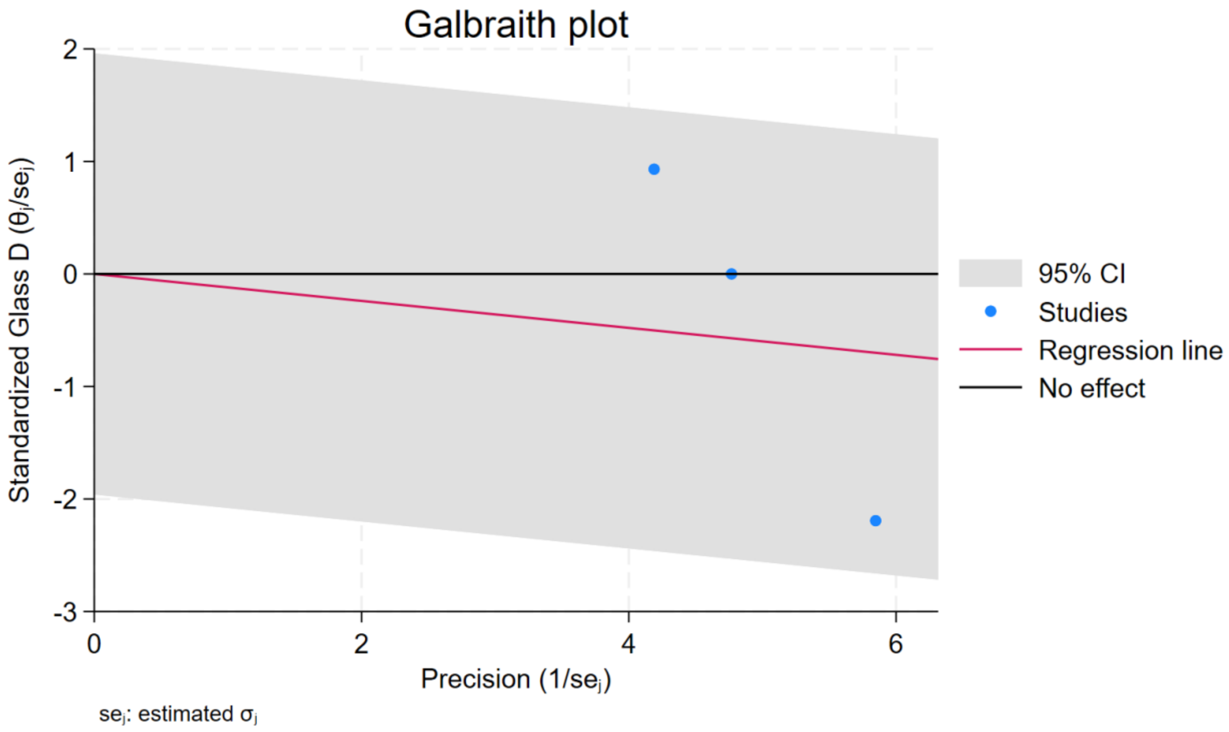

Trim-fill analysis and funnel plotting

The funnel plot for the endpoint of pH difference is shown in Figure S2-F2. The contoured funnel plot suggested possibility of small-study bias, but not publication bias. Two studies were imputed, both with projected statistically significant findings in the direction of lower pH in hypocalcaemic patients.

With only three studies, there was insufficient study *N* to enable formal funnel asymmetry analysis. However, the Glass D estimate of the observed+imputed studies had a 95% CI (-0.806 to 0.056) that continued to overlap the null finding.

Figure S2-F2. Contoured funnel plot for the endpoint of pH difference

Online Supplement 4.

The Incidence of Admission Ionised Hypocalcaemia in Paediatric Major Trauma – a Systematic Review and Meta-Analysis

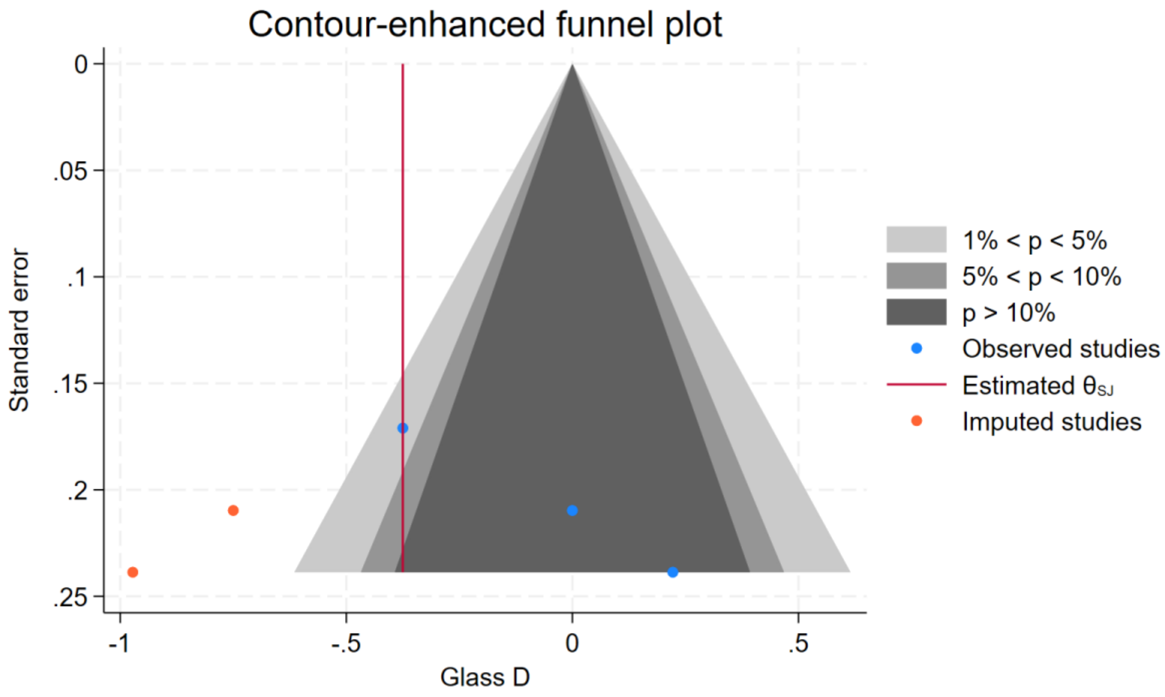

Cumulative meta-analysis

Cumulative meta-analysis for the endpoint was executed ordering studies based on increasing *n* (Figure S2-F3). The smallest *n* (Gimelraikh) commenced the cumulative meta-analysis with a non-significant indicator of higher pH in hypocalcaemics; larger studies both counterbalanced that finding's directionality but at a magnitude departure from null that was 50% or less of that of the smallest study. With increasing study *n* the effect estimate became closer to the null value.

Figure S2-F3. Cumulative analysis for the endpoint of pH difference

Online Supplement 4.

The Incidence of Admission Ionised Hypocalcaemia in Paediatric Major Trauma – a Systematic Review and Meta-Analysis

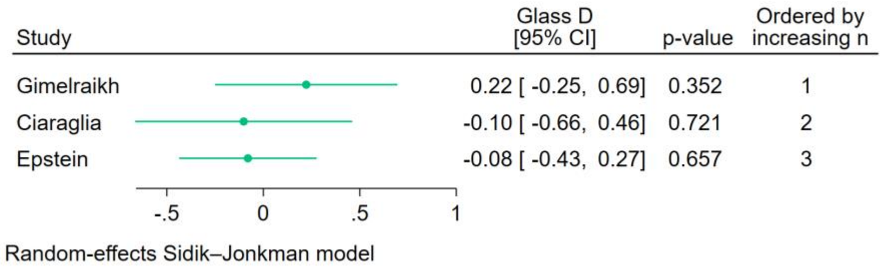

Omitted-study meta-analysis

To ascertain the degree to which individual study results executed inordinate leverage on the overall results, omitted-study (leave-one-out) meta-analysis was executed. The plot in Figure S2-F4 shows the effect estimate that would be generated if the named study were not included in the calculations. The actual overall effect estimate from all studies (*i.e.* as reported in main results) is depicted with a dashed line. The omitted-study analysis for the endpoint suggested that no individual study exerted undue leverage. Results did not change from the null conclusion (based on 95% CI) when any one of the three studies was excluded.

Figure S2-F4. Omitted-study plot for the endpoint of pH difference

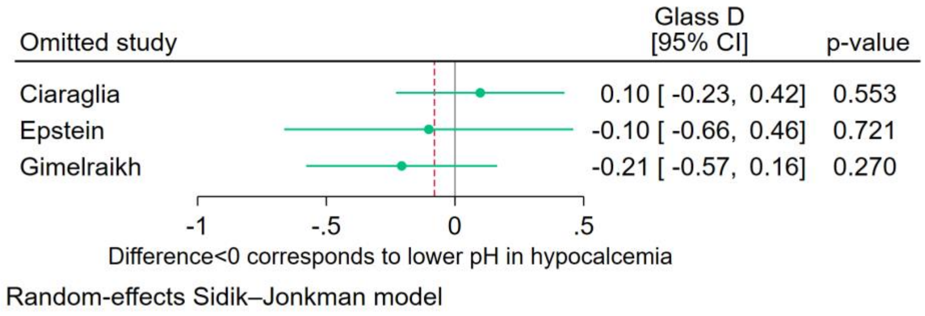

Supplement: S3 File — (PDF) [file pone.0303109.s004.pdf]
