# Supplementary material for: The incidence of admission ionised hypocalcaemia in paediatric major trauma—A systematic review and meta-analysis
Source: PLoS One. 2024 May 28;19(5):e0303109. doi: 10.1371/journal.pone.0303109 (PMC11132510; doi:10.1371/journal.pone.0303109)
Supplement: S4 File — (PDF) [file pone.0303109.s005.pdf]

Online Supplement 5.

The Incidence of Admission Ionised Hypocalcaemia in Paediatric Major Trauma – a Systematic Review and Meta-Analysis

<u>Trauma Team Activation Criteria for included studies</u>

Ciaraglia A, Lumbard D, Deschner B, et al. The effects of hypocalcemia in severely injured pediatric trauma patients. *J Trauma Acute Care Surg*. 2023;95(3):313-318. doi:10.1097/TA.0000000000003902

	Level 1-α	Level 1
Pediatric (age ≤17 y)	▪ Active airway assistance or respiratory distress<br>▪ Any O_2 saturation <90%<br>▪ Age-specific hypotension or absent distal pulses at any time<br>▪ Age-specific tachycardia at any time<br>▪ SI ≥1<br>▪ GCS ≤11 with trauma MOI<br>▪ Penetrating injury to head (or depressed skull fracture), neck, or torso<br>▪ Active external hemorrhage or ongoing transfusion<br>▪ Requiring vasopressors<br>▪ Transfer receiving blood, vasopressors, or any other Level 1-α criteria	▪ Any O_2 saturation <95%<br>▪ GCS ≤13 with trauma MOI<br>▪ Pelvic instability<br>▪ Chest wall instability or crepitus<br>▪ Acute paralysis, loss of sensation with suspected SCI<br>▪ Open fracture/amputation at or proximal to the wrist or ankle<br>▪ Penetrating injury at or proximal to the elbow or knee<br>▪ Pulseless extremity<br>▪ ≥2 Proximal long bone fractures<br>▪ Seatbelt sign to neck, chest, or abdomen<br>▪ Ejection from vehicle<br>▪ All burns ≥30% TBSA<br>▪ Smoke inhalation<br>▪ Transfers intubated or meeting Level 1 criteria

Epstein D, Ben Lulu H, Raz A, Bahouth H. Admission hypocalcemia in pediatric major trauma patients-An uncommon phenomenon associated with an increased need for urgent blood transfusion. *Transfusion*. 2022;62(7):1341-1346. doi:10.1111/trf.16936

(1) Glasgow Coma Scale (GSC) <9 or deteriorating by 2 points;

(2) Respiratory compromise/distress or patients intubated before admission;

(3) Hypotension- defined as systolic blood pressure (SBP)< [70mmHg+(2×age)];

(4) High-risk anatomical injury (e.g. penetrating injury to head, neck, torso, or extremities proximal to elbow/knee; multiple long bone fractures; pelvic fractures; amputation proximal to wrist/ankle; suspected spinal cord injury);

(5) High-risk mechanism of injury (e.g. high-speed crash; vehicle entrapment; ejection from vehicle; pedestrian/bicyclist struck by vehicle; fall from high);

(6) At the discretion of the emergency medical services or the emergency physician.

Gimelraikh Y, Berant R, Stein M, Berzon B, Epstein D, Samuel N. Early Hypocalcemia in Pediatric Major Trauma: A Retrospective Cohort Study. *Pediatr Emerg Care*. 2022;38(10):e1637-e1640. doi:10.1097/PEC.0000000000002719

(1) Glasgow Coma Scale (GSC) <9 or deteriorating by 2 points;

(2) Respiratory compromise/distress or patients intubated before admission;

(3) Hypotension- defined as systolic blood pressure (SBP)< [70mmHg+(2×age)];

(4) High-risk anatomical injury (e.g. penetrating injury to head, neck, torso, or extremities proximal to elbow/knee; multiple long bone fractures; pelvic fractures; amputation proximal to wrist/ankle; suspected spinal cord injury);

(5) High-risk mechanism of injury (e.g. high-speed crash; vehicle entrapment; ejection from vehicle; pedestrian/bicyclist struck by vehicle; fall from high);

(6) At the discretion of the emergency medical services or the emergency physician.
