# Supplementary material for: The incidence of admission ionised hypocalcaemia in paediatric major trauma—A systematic review and meta-analysis
Source: PLoS One. 2024 May 28;19(5):e0303109. doi: 10.1371/journal.pone.0303109 (PMC11132510; doi:10.1371/journal.pone.0303109)
Supplement: Attachment — Submitted filename: PONE-D-24-02104 Response to Reviewers.docx [file pone.0303109.s006.docx]

**Response to Reviewers**

Thank you to both reviewers for their valued comments, which have prompted further consideration of the topic.

Responses to both reviewers are detailed below. The authors would also be happy to respond to any further questions on request.

Reviewer 1

1. It looks like you have miscalculated the incidence of iHypoCa. Epstein's

article reports 21 patients with mild iHypoCa (1.0-1.1mmol/L) and 3 patients with severe

iHypoCa (<1.0mmol/L). This makes a total of 24 patients with iHypoCa <1.1mmol/L and not 21

as you have stated in your paper. On line 325 you correctly refer to the total number of 24.

This affects the overall incidence of iHypoCa. It probably doesn't change the conclusion, but

this error should be corrected.

**Thank you for identifying this. This has been corrected in the text and tables. Fortunately, the meta-analysis used the correct numbers.**

2. All 3 included articles report on the incidence of severe iHypoCa <1.0mmol/L. I would

recommend that you also include the incidence with this identical definition in your paper.

**Thank you. The incidence of severe iHypoCa for each study has now been included in Table 1. This is to demonstrate the incidence of severe iHypoCa whilst maintaining a focus on the variable definitions masking the true incidence and relevant cut-off values (line 405;line 503).**

3. (a)To support your statement that children may be more sensitive to iHypoCa (line 107), it is

suggested to include another reference in addition to Barcelona et al. This is because

Barcelona et al. only mention neonates as being more sensitive to citrate-induced iHypoCa,

which is a very limited group in the overall paediatric trauma population.

**Thank you. The following changes have been made, and the below references have been added.**

**Line 106 – 110**

**“In critically ill paediatric patients on the PICU hypocalcaemia has been observed to be independently associated with more severe organ dysfunction and more frequent amongst non-survivors compared to survivors. In trauma, children may be more sensitive to iHypoCa due to different injury kinematics and developmentally dynamic physiological responses to injury.”**

**Barcelona et al. reference removed.**

**Attard C, van der Straaten T, Karlaftis V, Monagle P, Ignjatovic V. Developmental hemostasis: age-specific differences in the levels of hemostatic proteins [published correction appears in J Thromb Haemost. 2019 Nov;17(11):1995]. J Thromb Haemost. 2013;11(10):1850-1854. doi:10.1111/jth.12372**

**Mullen S, Tolson A, Bouamra O, et al. Comparison of injury patterns and interventions between adolescent, adult and paediatric trauma cases: a cross-sectional review of TARN data. BMJ Open. 2023;13(5):e064101. Published 2023 May 9. doi:10.1136/bmjopen-2022-064101**

(b) Furthermore, Hobbs et al reported that transfusion-related iHypoCa was less prevalent in

children than in adults. You also mention an increased incidence of iHypoCa with older age.

Therefore, it remains unclear whether calcium imbalances are truly relevant for the paediatric

population and whether they should be studied differently from the adult trauma population.

**Thank you. This is a useful observation and we have made the following changes in the text and added the references to Hobbs et al:**

**Line 393-394**

**“This lower incidence in the paediatric population has also been observed for transfusion-related iHypoCa.”**

**Line 405-408**

**“As such, it remains unclear whether calcium imbalance in paediatric trauma should be considered different to the adult trauma population, and further studies would be useful to clarify relevance with different ages.”**

**Giancarelli A, Birrer KL, Alban RF, Hobbs BP, Liu-DeRyke X. Hypocalcemia in trauma patients receiving massive transfusion. *J Surg Res*. 2016;202(1):182-187. doi:10.1016/j.jss.2015.12.036**

**Livingston MH, Singh S, Merritt NH. Massive transfusion in paediatric and adolescent trauma patients: incidence, patient profile, and outcomes prior to a massive transfusion protocol. Injury. 2014;45(9):1301-1306. doi:10.1016/j.injury.2014.05.033**

4. In the first paragraph of the introduction (line 93), it is mentioned that treatment of

hypocalcemia is recommended to deliver effective trauma resuscitation. However, there is

currently no evidence to support a causal relationship between hypocalcemia and worse

outcomes, which would justify calcium supplementation. It is important to note that

association with worse outcomes does not necessarily mean that hypocalcemia is the cause.

Additionally, a similar relationship with iHyperCa was found in the adult population.

Similar, in this review in the paediatric population, only an association between iHypoCa and

haemodynamic instability and increased blood transfusion was found. The included articles

do not provide any evidence of a causal relationship. It is recommended to comment on this

in your article.

**Thank you. Your comment here is correct and the opening paragraph has been changed to reflect this.**

**Line 93-94**

**“Consequently, current guidelines recommend maintaining normal calcium levels in the bleeding trauma patient.”**

**This has also been addressed in the discussion:**

**Line 387-388**

**“The included articles do not provide evidence of a causal relationship between iHypoCa and adverse outcomes.”**

**Line 434-435**

**“Moreover, a recent systematic review of empirical calcium administration for adult patients in cardiac arrest has demonstrated harm”**

**Line 470-472**

**“Additionally, hypercalcaemia (iCa ≥ 1.30 mmol/l) has also been associated with mortality and adverse outcomes in adult trauma patients, suggesting that there is a parabolic association between calcium levels and adverse outcomes.”**

5. The difference in the incidence of iHypoCa between Ciaraglia's study and the other two is

remarkable, particularly considering the lower ISS in Ciaraglia's study. Although the

increased incidence of penetrating trauma and different trauma criteria may have

contributed, it is worth considering whether other factors may have played a role, like the

exclusion of iTBI. Epstein's study found that iTBI accounted for 17% of his trauma population. Can you comment on this?

**Thank you. We have referenced the exclusion of isolated head injury and uploaded the trauma team activation criteria for each study as a supplementary appendix.**

6. Line 359 should start with a capital letter.

**Thank you for identifying this. Line 359 now starts with a capital letter.**

7. In the paper, both 'hypocalcemia' and 'iHypoCa' are used. It is recommended to

consistently use 'iHypoCa'.

**Thank you for identifying this. Hypocalcaemia should now read as iHypoCa throughout the text. Ionised hypocalcaemia is still used for subheadings and tables. On review we also noticed a similar inconsistency with iNormoCa and normocalcaemia and have similarly endeavoured to correct this to iNormoCa throughout.**

Reviewer 2

1. You included a study in which the cutoff value for iCa was 1.16 mmol/L, which I think is

rather high. I think that 1mmol/L should be the standard for adults as well.

**Thank you. This is a useful and perceptive comment that further supports the review's rationale and conclusion that further research may be required to better de-lineate relevant cut-off values.**

**A cut-off of <1mmol/l is used in many studies. However, definitions are hugely variable across the literature, and, even amongst the small number of studies included in this review, some studies define <1mmol/l as severe hypocalcaemia, whilst Ciaraglia *et al.* define this as hypocalcaemia. The other two studies using higher cut-offs have still demonstrated relevant clinical associations. Additionally, including the study by Gimelraikh *et al.* is important as this cut-off of 1.16 mmol/l is a common laboratory reference value used, which may inform subsequent work related to mild or moderate hypocalcaemia.**

**The following changes have been made to the text and references added:**

**We have further separated incidence by iHypoCa < 1mmol/l in Table 3. Definitions and incidence of ionised hypocalcaemia in paediatric major trauma patients**

**Line 399-401**

**“The inclusion of these higher cut-off values is important as this reflects a common laboratory reference value and may also inform subsequent work related to mild or moderate hypocalcaemia.”**

**Line 405-408**

**“As such, it remains unclear whether calcium imbalance in paediatric trauma should be considered different to the adult trauma population, and further studies that are powered to detect age-related differences in a younger cohort would be useful to clarify relevance.”**

**Goyal A, Anastasopoulou C, Ngu M, Singh S. Hypocalcemia. In: *StatPearls*. Treasure Island (FL): StatPearls Publishing; October 15, 2023.**

**Bloom BM, Grundlingh J, Bestwick JP, Harris T. The role of venous blood gas in the emergency department: a systematic review and meta-analysis. *Eur J Emerg Med*. 2014;21(2):81-88. doi:10.1097/MEJ.0b013e32836437cf**

**Imamoto T, Sawano M. Effect of ionized calcium level on short-term prognosis in severe multiple trauma patients: a clinical study. Trauma Surg Acute Care Open. 2023;8(1):e001083. Published 2023 Jun 30. doi:10.1136/tsaco-2022-001083**

2. The inclusion criterion of trauma team activation is ambiguous. Shouldn't it be detailed?

**Thank you. We have now uploaded an online supplement detailing each study's trauma team activation criterion.**

3. You mentioned pH in the Discussion, but didn't you consider correcting the iCa value for

pH in the first place?

**Thank you. This is a useful observation, and I note that Imamoto *et al.* successfully accomplished this in their well-written study*.* An original data review may have achieved this. However, we felt that the complex and somewhat paradoxical relationship between iCa and pH in trauma may have risked underestimating the incidence of iHypoCa. This is because although alkalosis leads to intracellular calcium shifts, ischaemia and reperfusion injury can lead to pH-dependent lactate calcium binding suggesting that both alkalsosis and acidosis could theoretically lead to iHypoCa in trauma. Additionally, when applied to the clinical environment this is less relevant on the shop floor and may detract from the recognition and treatment of components of the “diamond of death”.**

**Hamroun A, Pekar JD, Lionet A, *et al*. Ionized calcium: analytical challenges and clinical relevance. J Lab Precis Med. 2020; 5: 22. doi:10.21037/jlpm-20-60**

**We hope this answers your question, commend your study, and have added the following to the text:**

**Line 487-490**

**“There is the potential for both acidosis and alkalosis to confound iCa levels; although a pH calcium correction formula can be used (with around 5% calcium level increase for each 0.1 pH unit decrease), this may risk underestimating iHypoCa and may be less relevant for interpretation in the clinical situation.”**

4. Shouldn't there be a slight mention of the Sidik-Jonkman approach? (This is not a

common approach.)

**Thank you. The text and reference below have been added.**

**Line 189 - 191**

**“The Sidik-Jonkman method was selected due to its superior performance for random effects meta-analysis, especially in small studies.”**

**IntHout J, Ioannidis JP, Borm GF. The Hartung-Knapp-Sidik-Jonkman method for random effects meta-analysis is straightforward and considerably outperforms the standard DerSimonian-Laird method. BMC Med Res Methodol. 2014;14:25. Published 2014 Feb 18. doi:10.1186/1471-2288-14-25**

5. You incorporated only one study in which a single head injury was excluded. Head trauma

increases the fibrinolytic system, which is prone to coagulopathy and, of course, affects iCa

levels in the adult population. What do you think about the possibility of underestimating the

results?

**Thank you. This is an astute observation. Perhaps paradoxically, the study excluding isolated severe head injury also had the lowest definition for hypocalcaemia and the highest incidence. Other factors, such as penetrating injury and trauma team activation, are relevant here and included in the text. There is a possibility that the exclusion of isolated head injuries may, therefore, have either under- or overestimated the results. Therefore, the following text and reference have been added.**

**Line 416 - 417**

**“Head injuries are recognised to increase the fibrinolytic system and are prone to coagulopathy; the true effect of excluding these isolated injuries in Ciaraglia et al.’s study is therefore unclear.”**

**Peng S, Lv K. The role of fibrinogen in traumatic brain injury: from molecular pathological mechanisms to clinical management. Eur J Trauma Emerg Surg. 2023;49(4):1665-1672. doi:10.1007/s00068-022-02084-w**

6. With regard to pH, we have to say that statistical heterogeneity is SUBSTANTIAL

heterogenenity. What do you think about the possibility of pH direct confounding for iCa?

**Thank you. I hope we have answered this above in question 3.**
